# Investigating Leptomeningeal Anastomoses’ Role in Collateral Blood Flow using SPECT and 4D Flow MRI

**DOI:** 10.1007/s10439-026-04003-1

**Published:** 2026-02-20

**Authors:** Chi Hang To, Marie Oshima, Shigeki Yamada

**Affiliations:** 1 School of Engineering, The University of Tokyo, 7-3-1 Hongo, Bunkyo-ku, 113-8656 Tokyo Japan; 2 Graduate School of Medical Sciences, Nagoya City University, 1 Kawasumi, Mizuho-cho, Mizuho-ku, Nagoya, 467-8601 Aichi Japan

**Keywords:** Leptomeningeal anastomoses, Cerebral collateral circulation, Patient-specific modeling, 4D Flow MRI, Single-Photon Emission Computed Tomography, Genetic algorithm optimization

## Abstract

Leptomeningeal anastomoses (LMAs) are vital components of cerebral collateral circulation, but their small size and inter-patient variability hinder direct quantitative assessment in patient-specific cases. This study investigates the functional role of LMAs and their vessel-level interactions with the cerebral vasculature, using patient-specific flow data and accounting for uncertainty in peripheral vessel anatomy. Synthetic vascular trees were generated via a stochastic, anatomically informed sampling process. Assuming that discrepancies between 4D Flow MRI and Single-Photon Emission Computed Tomography (SPECT) are attributable to LMA-mediated redistribution, LMA configurations were optimized using an island genetic algorithm (IGA) to minimize residuals between simulated distal flows and SPECT-derived perfusion data. The resulting configurations successfully recreated physiologically plausible collateral patterns across four clinical scenarios, encompassing mild to severe, asymmetric, and symptomatic unilateral stenosis.

## Introduction

During ischemia, cerebral blood flow redistributes from unaffected to compromised regions through collateral pathways [1], preserving the penumbra and delaying irreversible damage [2, 3]. Timely flow restoration is critical, as neuron loss becomes permanent within hours [4]. This is exacerbated by impaired autoregulation in early ischemia [3]. Six vascular regions—anterior, middle, and posterior cerebral arteries (ACA, MCA, PCA) in each hemisphere—are linked by two main collateral systems: the Circle of Willis (CoW) and leptomeningeal anastomoses (LMAs) [5]. LMAs can sustain tissue hours after major artery occlusion and are associated with reduced infarct volume and improved recovery [6–8]. Their variable anatomy and sub-millimeter caliber hinder quantification [9, 10].

Imaging resolution limits and angiography’s invasiveness have historically constrained LMA studies [11–13]. Recent advances in high-resolution imaging and computational methods, including radiomic analysis of perfusion scans, now permit improved collateral assessment [14]. LMAs are implicated in various cerebrovascular conditions, with effectiveness influencing both infarct size and recovery [15]. In internal carotid artery (ICA) stenosis, collateral development increases with severity [16, 17]; ICA stenosis is therefore the focus here, due to its broader perfusion impact, more complex redistribution patterns, and diagnostic accessibility via ultrasound compared to MCA occlusion.

Two modeling strategies dominate: lumped parameter models, which represent territories as compartments connected by resistors [18, 19], and explicit vasculature models, which preserve branching topology and geometry. While lumped models are efficient, they rely on generalized conductances and offer limited LMA-specific insight. Explicit models better capture structural constraints; for example, Epp et al. [20] used micro-scale reconstructions to show that passive pressure gradients, rather than active dilation, drive LMA-mediated redistribution. Human-specific implementations such as Ii et al. [21] and Otani et al. [22] combine image-derived CoW geometries with synthetic cortical vasculature. However, the smallest vessels modeled remain above clinical imaging resolution, and computational generation via constrained constructive optimization (CCO) can take over an hour [21], limiting iterative simulation.

This study aims to characterize how leptomeningeal anastomoses redistribute blood flow between cortical territories under physiological constraints using patient-derived flow data. To achieve this aim, we establish a modeling framework with three objectives (1) to construct anatomically plausible cortical vascular networks that incorporate variability in branching structure and vessel caliber; (2) to quantify the distal flow distribution these networks produce under physiological inlet conditions; and (3) to infer the functional role of LMAs by solving an inverse problem that identifies the collateral patterns most consistent with patient-specific 4D Flow MRI and SPECT measurements.

## Materials and Methods

**Fig. 1:**
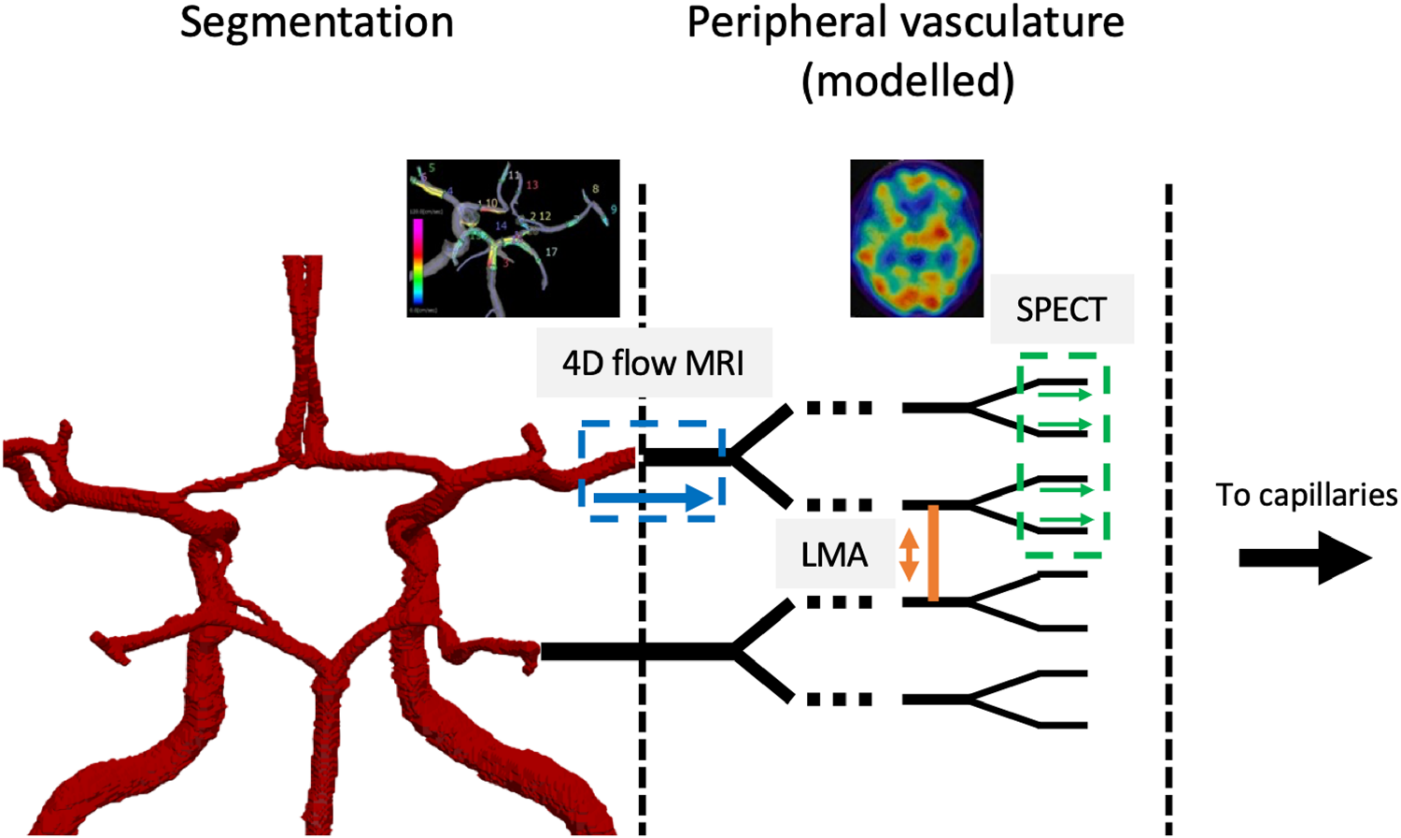
Circle of Willis (CoW) and schematic representation of peripheral vasculature. 4D Flow MRI and SPECT images are shown, with dotted rings indicating the regions where flow rates are measured. The leptomeningeal anastomoses (LMA) are highlighted to demonstrate their role in accounting for the differences between the two measurements within each vascular region.

Figure 1 gives a schematic view that assist in understanding the overall workflow of the study. In the first stage, synthetic vascular networks are generated for the six cortical regions by sampling bifurcation patterns, vessel radii, and tree structures from cadaver-derived statistics, providing anatomically plausible geometries with explicit support for LMA placement and controlled variability. In the second stage, blood flow simulations are performed to compute distal flow rates within each vascular region, establishing the functional baseline that LMAs act upon. In the final stage, an inverse-problem formulation is solved using an island genetic algorithm, which optimizes both the number and the location of LMAs such that the resulting collateral redistribution matches patient-derived distal flow rates obtained from 4D Flow MRI and SPECT. These three stages—vascular generation, flow simulation, and optimization—structure the Methods section, where each component is described in detail.

### Data Acquisition

Patient-specific data from imaging were used to inform the model and guide optimization of leptomeningeal anastomoses (LMAs). The three primary data types are summarized in Table 1.

Radii of the Circle of Willis (CoW) efferent arteries, which serve as model inlets, were extracted from segmented CT angiography or TOF-MRA datasets using the V-Modeler software [23, 24]. The CT datasets were acquired on Aquilion ONE (Canon Medical Systems) and SOMATOM Definition Flash (Siemens Healthineers) scanners, while the MR angiography dataset was obtained on a DISCOVERY MR750w (GE Healthcare) system. Vessel radii were computed by averaging cross-sectional measurements along smoothed centerlines to provide representative inlet dimensions for synthetic vasculature generation.

SPECT data were acquired using a Siemens E-Cam rotating gamma camera (64×64 matrix, 9-mm FWHM) [25]. Regional flow proportions were obtained by registering a standard vascular template to each patient’s SPECT via Large Deformation Diffeomorphic Metric Mapping. Voxel intensities within segmented regions determined the relative flow proportions. To account for differences in total cerebral blood flow measured by SPECT and 4D Flow MRI, regional SPECT flows were scaled to match the total flow rate at CoW inlets, computed as the sum of left and right internal carotid and basilar artery flows measured by 4D Flow MRI.

4D Flow MRI measurements, acquired on a 64-channel 3T MAGNETOM Skyra system (Siemens), were also scaled to the total flow at CoW inlets to compensate for decreased accuracy in smaller arteries (diameter <3 mm). The scaled MRI flows were used to prescribe boundary conditions in the model.

**Table 1:** Summary of imaging data and their roles in the modeling framework

**Data Type**	**Source/Acquisition**	**Role in Model**
CT/MRA	CT: Aquilion ONE (Canon Medical Systems) and SOMATOM Definition Flash (Siemens Healthineers); MRA: DISCOVERY MR750w (GE Healthcare)	Radii of CoW efferent arteries for synthetic vasculature generation
SPECT	Siemens E-Cam rotating gamma camera (64×64 matrix, 9-mm FWHM) [25]	Regional distal perfusion fractions used to guide LMA optimization
4D Flow MRI	3 T MAGNETOM Skyra (Siemens), 64-channel coil	Proximal inflow distribution at CoW outlets for boundary condition assignment

### Vascular Model

The vascular model is constructed separately for each of the six vascular regions and organized into three anatomical levels:

- **Level 1**: Main trunks crossing the brain posteriorly (e.g., superior and inferior MCA trunks).
- **Level 2**: Perforating branches extending orthogonally from level 1 arteries (e.g., Orbitofrontal and Angular arteries).
- **Level 3**: Cortical arterioles branching from level 2 arteries to supply cortex; LMAs connect these arterioles.

**Fig. 2:**
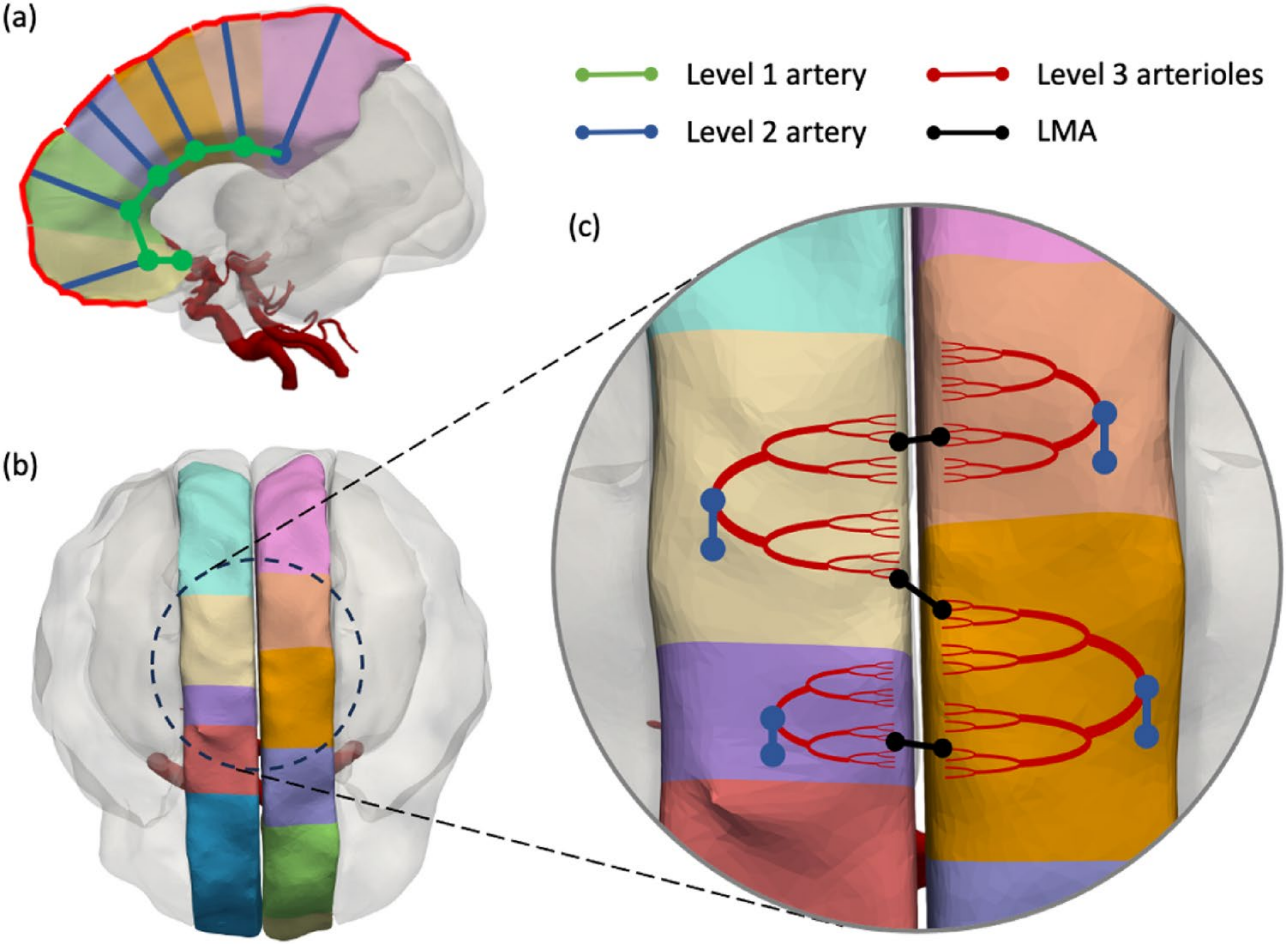
**a** Side view illustrating three hierarchical arterial levels: level 1 arteries span anterior–posteriorly, level 2 arteries branch perpendicularly toward the cortical surface, and level 3 arterioles are embedded in the cortex. **b** Top view of governing regions supplied by level 2 arteries in the anterior cerebral artery (ACA) territory of both hemispheres. **c** A close-up highlights leptomeningeal anastomosis (LMA) connections between level 3 arterioles across adjacent regions.

Figure 2a shows the vascular model linked to the CoW, highlighting its hierarchical structure. The spatial layout of the three levels is depicted, with the number of level 2 arteries varying across regions and hemispheres due to stochastic sampling, detailed below.

Level 1 and 2 arteries (diameter $>1$ mm) are sometimes derived from patient imaging but often with uncertainty or incomplete detail. Our method addresses this by considering a range of vascular configurations, serving as uncertainty analysis in blood flow simulations. Preserving the branching structure and radius distribution of level 2 arteries is critical for meaningful simulation, as applying Murray’s law directly from CoW efferents would be insufficient to maintain this anatomical fidelity.

Assuming cortical area supplied by a perforating artery is proportional to its flow, we estimate each level 2 artery’s territory. As these arteries penetrate cortex perpendicularly, their positions along level 1 arteries correspond to cortical-region layout. The cortex can then be divided into eight longitudinal strips representing major arterial territories: LPCA, LMCA inferior, LMCA superior, LACA, RACA, RMCA superior, RMCA inferior, and RPCA, with the MCA splitting into superior and inferior trunks [26], each supplying distinct, minimally overlapping cortical areas. These strips denote the adjacency of cortical regions, and constrains the location of LMAs.

Figure 2b and c illustrates this spatial arrangement. Cortical regions align longitudinally along the anterior–posterior axis, following level 1 artery paths. Region sizes are proportional to the flow through their respective level 2 arteries.

To generate diverse vascular models, we use the Independent Marginals (IM) method, a simple and efficient synthetic data technique. Although more advanced methods exist [27], IM suits the distributional (not patient-specific) nature of our data. More complex methods may be adopted when detailed inputs become available. Parameters are derived from key anatomical data [26, 28, 29].

**Fig. 3:**
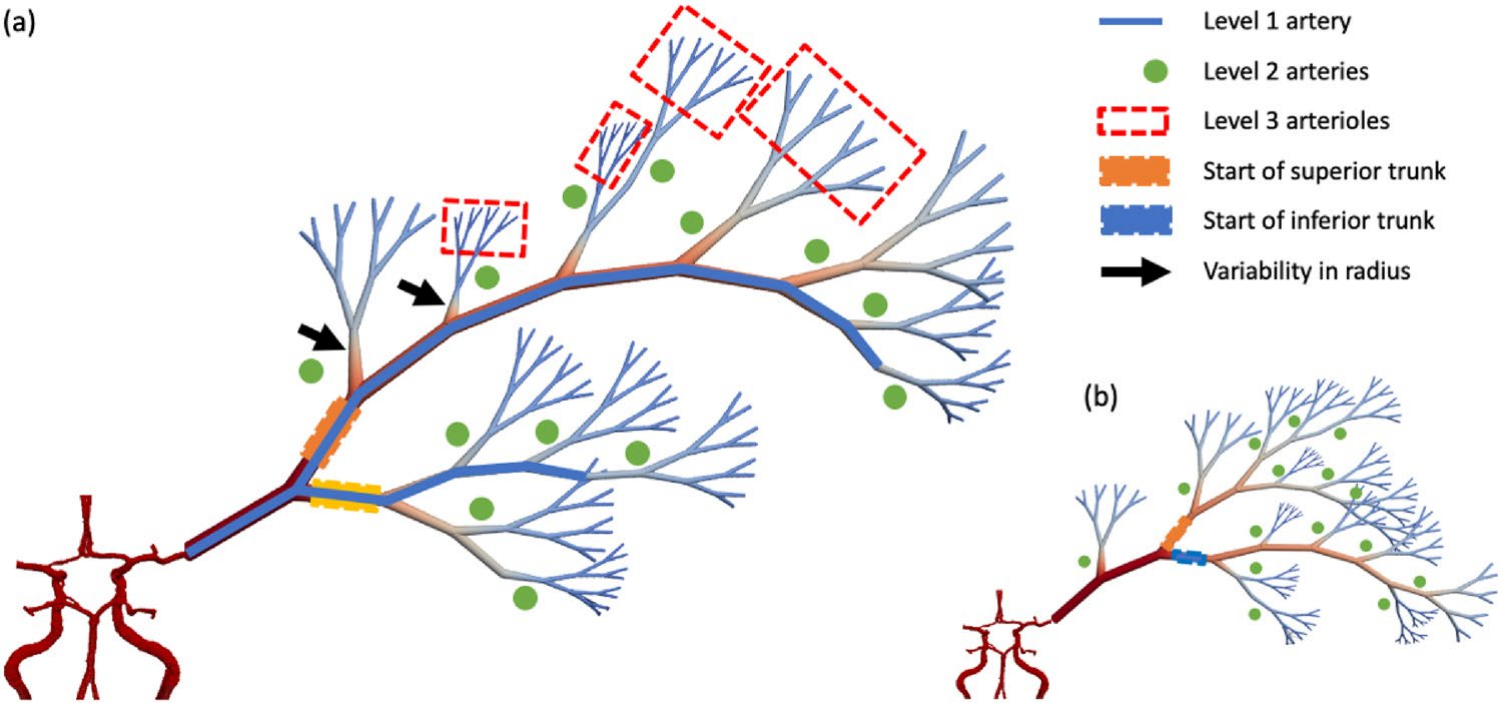
**a** Example of a sampled middle cerebral artery (MCA) model, illustrating the 3 levels of arteries common across all models. **b** Another sampled MCA model highlighting key sources of structural variability: the number and configuration of level 2 arteries relative to the main trunks, variations in artery radius, and the presence and positioning of commonly shared stems.

A probabilistic generative process, guided by frequency data, constructs anatomically plausible branching under uncertainty. Figure 3 shows example results: panel (a) illustrates a sampled MCA model, while panel (b) highlights variability in level 2 artery count, arrangement, diameter, and stem-sharing. The model generation follows these steps:

1. **Presence Sampling:** For each anatomically defined level 2 artery, the number of instances $N_i$ is sampled:
  If the artery does **not fenestrate**, $N_i \in \{0,1\}$, based on reported frequency.
  If the artery **can fenestrate**, $N_i \in \mathbb {N}_0$, sampled from a Poisson distribution using literature-reported means and ranges [26, 29].
2. **Trunk Assignment:** Each sampled artery is assigned a main trunk via a categorical distribution, reflecting origin probabilities from literature [26, 28, 29].
3. **Stem-Sharing Assignment:** Conditional on trunk assignment, the model determines whether arteries share a common stem, based on discrete probabilities from anatomical studies.

To model arterial radii, level 2 artery diameters $r_{i,\text {original}}$ are sampled from a distribution with mean $\mu$ and bounds $[r_{\min }, r_{\max }]$.

A uniform scaling factor $\lambda$ is applied to ensure the distribution conforms to Murray’s law, which reliably predicts vessel radii [30]. To satisfy it while preserving proportionality between branches, all radii are uniformly scaled such that

1 $$\begin{aligned} \sum _i r_{i,\text {scaled}}^3 = r_{\text {inlet}}^3 , \end{aligned}$$

where $r_{\text {inlet}}$ is the inlet radius measured from imaging. This value is sampled within a $\pm 10\%$ uncertainty range for each vascular region and patient. With level 2 artery radii fixed, level 1 segment radii are computed recursively from distal to proximal using Murray’s law. Level 3 arterioles are generated likewise, branching symmetrically until diameters fall below 50 µm [31]. The main trunk length is assumed constant, as it has minimal effect on flow distribution to level 2 arteries. For each vascular region, we assign a total trunk length $\mathcal {L}_{\text {trunk}}$ from anatomical references, with each level 1 segment set to length $\mathcal {L}_{\text {trunk}} / n_m$ [32, 33]. Lengths of level 2 and 3 segments are determined using a constant length-to-radius ratio of 16.4, based on estimates from multiple studies [34–36].

Duvernoy et al. [31] identified two distinct types of LMA: large end-to-end and small adjacent-vessel anastomoses. Otani et al. [22] found that pial LMAs primarily affect localized zones, with only one-tenth the impact of larger ones. This study therefore targets the latter. Each LMA is indexed and identified as

2 $$\begin{aligned} \ell = \left( (m^{(a)}, i^{(a)}, d^{(a)}),\; (m^{(b)}, i^{(b)}, d^{(b)}) \right) , \end{aligned}$$

where (*m*, *i*, *d*) denotes vascular region, parent artery index, and bifurcation depth. Arterioles sharing the same triplet are treated as equivalent, abstracting away spatial geometry.

Figure 2c illustrates this framework. Level 2 arteries terminate at the cortical surface, from which level 3 arterioles emerge to supply cortex. LMAs link arterioles from neighboring cortical regions at specified bifurcation levels. While the diagram assumes unidirectional branching for clarity, real patterns are multidirectional, and LMAs may connect arterioles of different depths.

In this study, cortical perfusion territories were represented using a simplified one-dimensional anterior–posterior layout. Each vascular region was modeled as a continuous anterior–posterior strip, segmented into discrete units, with each unit corresponding to a level 2 cortical artery. This abstraction preserves the natural ordering of cortical branches while avoiding the need to explicitly model the three-dimensional cortical surface. Under baseline conditions, we assumed a uniform target perfusion rate across the cortex. Flow simulations were then performed with each synthetic vascular tree to determine the relative flow entering each cortical branch. The size of each segment was assigned in proportion to the simulated flow of its corresponding cortical artery: branches carrying higher flow were allocated larger cortical territories, whereas smaller branches governed proportionally smaller regions. Functionally, this scaling also preserves a physiologically intuitive property: flow-dominant branches generate larger cortical regions, resulting in greater adjacency with neighboring territories and, consequently, a higher likelihood of leptomeningeal collateralization.

Shared boundaries between vascular regions define possible LMA locations. In this model, the ACA is adjacent to the superior MCA territory, the PCA to the inferior MCA, and the left and right ACAs are adjacent. Additionally, the posterior 30% of the ACA territory borders the posterior 30% of the PCA territory through watershed zones in the posterior precuneus, as described by [37] and [9]. The LMA radius is defined as the average of the radii at both ends. Arteriole radius depends on depth and its parent level 2 artery’s radius:

3 $$\begin{aligned} r_d = r_{d=0} \left( \frac{1}{2^{1/3}} \right) ^d, \end{aligned}$$

No literature directly reports LMA length-to-radius ratios. We estimate this as 2.5, based on expected proximity of distally connected arterioles, supported by observations from Helthuis et al. [34].

### Fluid Simulation

The vasculature is modeled as a network of nodes connected by cylindrical rigid edges. Each segment’s resistance is calculated using Poiseuille’s law:

4 $$\begin{aligned} R = \frac{8 \mu L}{\pi r^4}, \end{aligned}$$

where $\mu$ is the blood viscosity, $L$ is the vessel length, and $r$ is the vessel radius.

Homogeneous cortical arteriole branches from a single vessel are merged into equivalent resistances using series and parallel combinations, enhancing computational efficiency for iterative LMA optimization. Introducing an LMA adds a node at the connection point, with upstream and downstream resistances recalculated accordingly.

A 0D steady-state flow model is used, assuming blood is an incompressible Newtonian fluid and that pressure and flow rate derivatives along each segment are negligible. Mass conservation is enforced at internal nodes:

5 $$\begin{aligned} \sum _{k} Q_{jk} = 0, \end{aligned}$$

where the sum is over nodes $k$ connected to node $j$. Flow between nodes $j$ and $k$ is thus given by

6 $$\begin{aligned} Q_{jk} = \frac{p_j - p_k}{R_{jk}}, \end{aligned}$$

where $p_j$ and $p_k$ are pressures at nodes $j$ and $k$, and $R_{jk}$ is the hydraulic resistance between them.

Boundary conditions prescribe flow rates at six inlet nodes (left/right ACA, MCA, PCA) from patient-specific 4D Flow MRI, and apply a fixed pressure of 50 mmHg at outlet nodes (level 3 arteriole terminals), representing typical cortical pial pressure. These are incorporated by modifying system matrices.

The resulting sparse linear system is solved directly using compressed sparse matrix methods, yielding pressures and flows across all nodes and segments, including LMAs. Regional distal flow is computed by summing outlet flows within each vascular territory.

We acknowledge the model approximates LMA-induced redistribution as uniform across arteries at the same cortical depth branching from a level 2 artery. Further analysis indicates that pressure differences among arteries at the same depth minimally affect results, justifying this assumption applied consistently to all LMA connections.

### Optimization of LMA Configuration

This section identifies LMA configurations explaining flow redistribution in case studies. Flow redistribution is inferred from differences between 4D Flow MRI inlet flows and SPECT-derived regional perfusion. Starting from six vascular trees without LMAs, the aim is to determine connections that reproduce the measured redistribution.

**Fig. 4:**
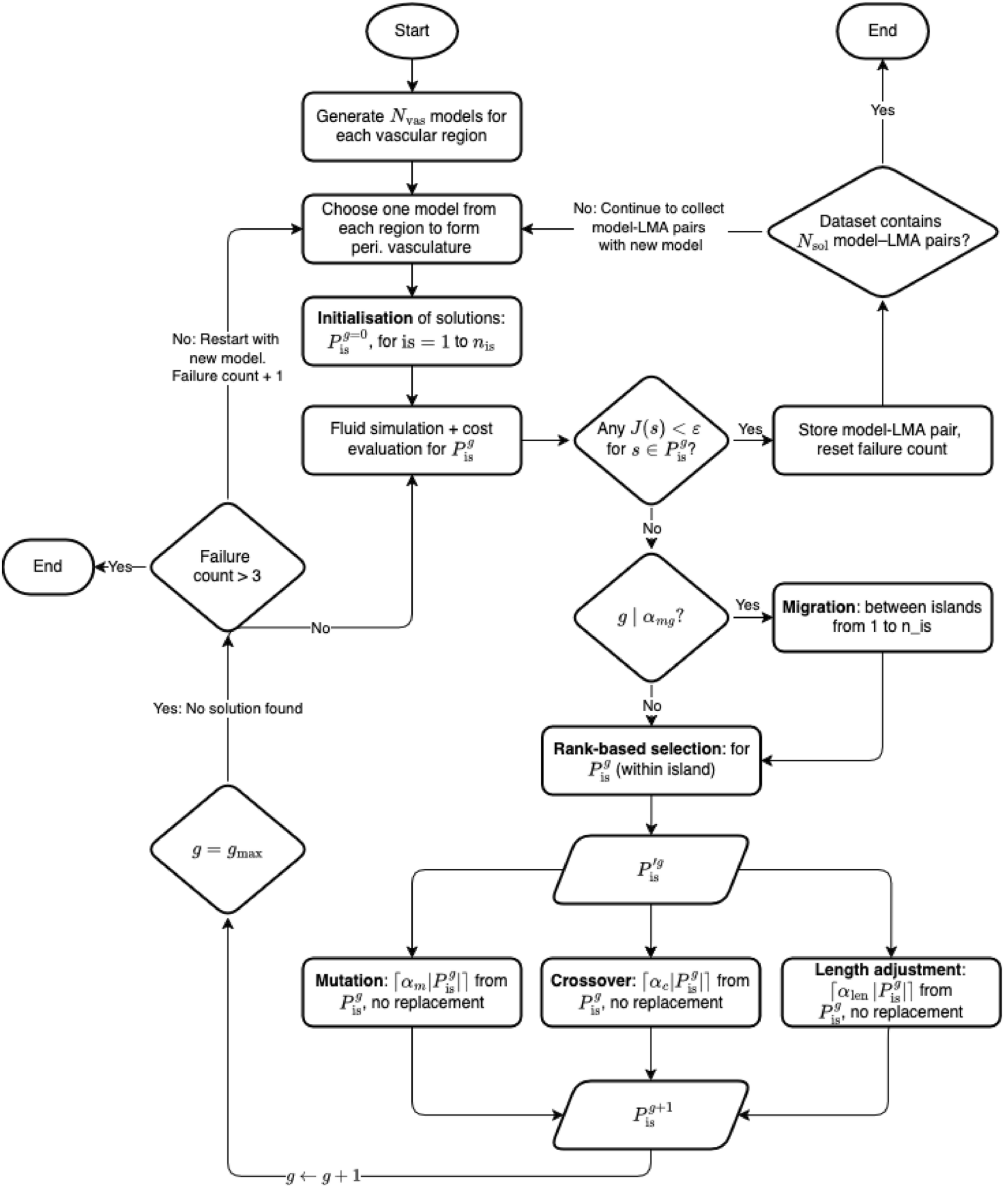
Flowchart of the optimization algorithm. Two main iterative loops are depicted. The outer loop begins after generating vascular models and involves constructing a peripheral vasculature composed of six sampled vascular trees. For each constructed vasculature, the inner loop optimizes the LMA configuration by iteratively evaluating the cost function, exploiting promising solutions, and exploring new candidates. This nested optimization framework seeks the LMA configuration that best explains the observed flow redistribution.

The search for optimal LMA configurations forms a large combinatorial space, unsuitable for exhaustive enumeration, motivating the use of an Island Genetic Algorithm (IGA). The algorithm evaluates candidate configurations by comparing simulated distal flows with SPECT targets and iteratively refines the population through the pipeline in Fig. 4. Customizations include rank-based selection, uniform crossover, and length-adjustment operators that vary the number of LMAs per candidate solution, which are detailed in the following subsections. GA parameters are tuned via Bayesian optimization to balance diversity, solution stability, and efficiency.

A solution $s$ is a set of $L_s$ LMAs:

7 $$\begin{aligned} s = \{ \ell _l \mid l = 1, 2, \dots , L_s \}, \end{aligned}$$

where $\ell _l$ is the position of the $l$-th LMA. The number of LMAs $L_s$ may vary between solutions.

The cost function is the root mean square difference between computed and SPECT-derived flows across all six vascular regions:

8 $$\begin{aligned} J = \sqrt{\frac{1}{M} \sum _{m=1}^{M} \left( Q_{\textrm{Co}, m} - Q_{\textrm{SP}, m} \right) ^2} \end{aligned}$$

Constraints: LMAs must connect different vascular regions within anatomically reported pairs [9, 22, 37] (ACA–superior MCA, PCA–inferior MCA, ACA–PCA, inter-hemispheric ACA, and left–right PCA or ACA via pericallosal anastomoses). Eligible arterioles pairs for connection have radius differences $\le 0.1$ mm, originate from level 2 arteries supplying spatially adjacent cortical regions, and are at least four bifurcations downstream from the cortical branch. No arteriole may connect to multiple regions; each eligible node pair allows at most one LMA.

A solution is valid if

9 $$\begin{aligned} \left| Q_{\textrm{Co}, m} - Q_{\textrm{SP}, m} \right| \le 0.05 \cdot Q_{\textrm{SP}, m}, \quad \forall m = 1, 2, \dots , M, \end{aligned}$$

with $$M = 6$$.

Wall shear stress for an LMA between nodes $j$ and $k$ is

10 $$\begin{aligned} \tau _{jk} = \left| \frac{4 \mu Q_{jk}}{\pi r_{jk}^3} \right| , \end{aligned}$$

and must satisfy

where $\mu$ is the dynamic viscosity of blood, $Q_{jk}$ is the flow rate, and $r_{jk}$ is the radius of the LMA. Every LMA must satisfy

11 $$\begin{aligned} \tau _l \ge \tau _{\textrm{min}}, \quad \tau _{\textrm{min}} = 2.5\ \textrm{Pa}, \end{aligned}$$

based on cerebral arteriole measurements [38].

#### Initialization

The optimization begins by generating 2000 vascular trees per region. From this pool, one tree per region is randomly selected to form a fixed peripheral vasculature for LMA optimization. A multi-island genetic algorithm (GA) with $n_{\text {is}} = 3$ independent populations (islands) is employed. Each island evolves independently with a population size of 118 candidate solutions per generation. To maintain diversity and avoid premature convergence, periodic migration exchanges occur every $\alpha _{\text {mg}} = 5$ generations, where each island shares a fraction $\beta _{\text {mg}} = 0.155$ of its population with other islands. Solutions initialize with $\mathcal {I} = 50$ LMAs, reflecting a sparse, biologically plausible starting point that enables demand-driven collateral formation. Islands run in parallel on separate processing cores.

#### Rank-based Selection Algorithm

Selection pressure is applied via a rank-based exponential scheme. Each solution $s_n$ is assigned a rank $\kappa _n \in \{1, 2, \dots , S\}$, where $1$ corresponds to the worst and $118$ to the best solution. Selection probabilities follow

12 $$\begin{aligned} \mathcal {P}_n = \frac{\kappa _n^{\epsilon }}{\sum _{h=1}^S \kappa _h^{\epsilon }}, \end{aligned}$$

where $\epsilon = 2.75$ controls selection pressure. Selection is performed $S$ times per generation with replacement to form the parent pool for the next generation. To address reduced sensitivity when costs converge, $\epsilon$ is dynamically increased if a promising solution is near feasibility, defined as having residuals within 15% of the SPECT-derived targets across all regions:

13 $$\begin{aligned} \epsilon _{\text {adj}} = {\left\{ \begin{array}{ll} \epsilon \left( 1 + 0.2 \cdot \dfrac{g}{g_{\text {max}}} \right) , & \text {if } \exists s \in P_{\text {is}}^{g} \text { such that} \\ & \quad \forall m, \left| Q_{\text {Co}, m}(s) - Q_{\text {SP}, m} \right| \le 0.15 \cdot Q_{\text {SP}, m}, \\ \epsilon , & \text {otherwise}, \end{array}\right. } \end{aligned}$$

where $g$ is the current generation, $g_{\text {max}}$ the total number of generations, and $m$ indexes vascular regions. $Q_{\text {Co}, m}(s)$ is the computed distal flow in region $m$ for solution $s$, and $Q_{\text {SP}, m}$ is the SPECT target flow. After selection, a parent pool $P'$ is formed to produce the next generation via crossover, mutation, and length adjustment.

#### Crossover, Mutation, Length Adjustment, and Migration

A modified uniform crossover explores the solution space. The crossover ratio $\alpha _c = 0.773$ determines the number of offspring produced relative to population size $S$. Given two parent solutions

14 $$\begin{aligned} s^{(a)} = \{ \ell ^{(a)}_1, \dots , \ell ^{(a)}_{L_a} \}, \quad s^{(b)} = \{ \ell ^{(b)}_1, \dots , \ell ^{(b)}_{L_b} \} \end{aligned}$$

define $L_{\min } = \min (L_a, L_b)$ and $L_{\max } = \max (L_a, L_b)$. Two children $s'^{(a)}$ and $s'^{(b)}$ are created as follows. For $l = 1, \dots , L_{\min }$, draw $X_l \sim \text {Uniform}(0,1)$ and set

15 $$\begin{aligned} \ell _l^{(a')} = {\left\{ \begin{array}{ll} \ell _l^{(b)}, & \text {if } X_l< 0.5 \\ \ell _l^{(a)}, & \text {otherwise} \end{array}\right. }, \quad \ell _l^{(b')} = {\left\{ \begin{array}{ll} \ell _l^{(a)}, & \text {if } X_l < 0.5 \\ \ell _l^{(b)}, & \text {otherwise} \end{array}\right. } \end{aligned}$$

For $l = L_{\min }+1, \dots , L_{\max }$, remaining LMAs from the longer parent are assigned randomly to either child:

16 $$\begin{aligned} \ell _l \rightarrow {\left\{ \begin{array}{ll} s'^{(a)}, & \text {if } X_l < 0.5 \\ s'^{(b)}, & \text {otherwise} \end{array}\right. } \end{aligned}$$

The mutation ratio $\alpha _m = 0.05$ defines the number of mutated offspring. For each $\ell _l \in s$, draw $X_l' \sim \text {Uniform}(0,1)$. With mutation rate $\beta _m = 0.11$, apply

17 $$\begin{aligned} \ell _l \rightarrow {\left\{ \begin{array}{ll} \ell _l', & \text {if } X_l' < \beta _m \\ \ell _l, & \text {otherwise} \end{array}\right. }, \end{aligned}$$

where $\ell _l'$ is a new valid LMA.

Length adjustment, inspired by Zhou et al. [39], is applied to a fraction $\alpha _{\text {len}} = 0.117$ of solutions. Let $J_{\text {long}}$ and $J_{\text {short}}$ be average costs for longer and shorter solutions than $L_s$. If $J_{\text {long}}> J_{\text {short}}$, add 1–6 LMAs; otherwise, remove 1–6 LMAs. If over 20% of the population shares the same length $L$, additional random adjustments (adding or removing 1 to 6 LMAs) are applied to promote diversity.

Each parent undergoes exactly one operation per generation:

18 $$\begin{aligned} \alpha _c + \alpha _m + \alpha _{\text {len}} = 1 \end{aligned}.$$

#### Termination

The optimization proceeds until either a satisfactory solution is identified or the maximum number of generations ($g_{\text {max}} = 300$) is reached. For each completed optimization run, the satisfactory LMA configuration and its associated peripheral vasculature are recorded, after which the six underlying vascular trees are returned to their respective pools for reuse. This process is repeated until $N_{\text {sol}} = 200$ distinct LMA-configuration sets have been collected.

It should be noted that the optimization algorithm itself is capable of producing far more than 200 unique configurations; the value of 200 is a deliberately chosen sample size. In the Results section, we demonstrate, via a sensitivity analysis, that increasing $N_{\text {sol}}$ does not materially alter the statistical behavior of flow redistribution, thereby justifying that 200 solution sets are sufficient for functional characterization of the networks.

Table 2 summarizes the primary variables used in the algorithm, including population sizes, migration settings, and crossover/mutation parameters. On the other hand, Table 3 lists the resulting outputs from the algorithm, including the optimized LMA configurations and the fixed peripheral vasculature employed in each run.

**Table 2:** Optimization algorithm variables

**Variable**	**Value**	**Description**
$N_{\text {is}}$	3	Number of islands (independent populations)
*S*	118	Number of solutions per island per generation
$\alpha _{\text {mg}}$	5	Generations between migrations
$\beta _{\text {mg}}$	0.155	Fraction of solutions exchanged between islands
$\mathcal {I}$	50	Initial number of LMAs in each solution
$g_{\text {max}}$	300	Maximum number of generations per optimization run
$N_{\text {sol}}$	200	Number of valid solutions to collect per patient
$\alpha _c$	0.773	Fraction of offspring produced via crossover
$\alpha _m$	0.05	Fraction of offspring subjected to mutation per generation
$\beta _m$	0.11	Probability that a given LMA is mutated
$\alpha _{\text {len}}$	0.117	Fraction of population adjusted for LMA quantity per generation

**Table 3:** Outputs of the optimization algorithm

**Output**	**Description**
Optimized LMA configuration	Locations and connections of LMAs for each solution
Peripheral vasculature	Fixed vascular trees used for LMA optimization

## Results

Four patient cases (Table 4) represent diverse clinical presentations and stenosis patterns, including unilateral and bilateral disease of varying severity. For each case, 200 vasculature–LMA configurations were optimized using patient-specific Circle of Willis (CoW) radii and fluid simulations to replicate SPECT-measured distal flows.

**Table 4:** Summary of patient characteristics

**Case ID**	**Age (years)**	**Gender**	**Symptomatic**	**Stenosis (Severity)**
CEA12	68	Male	Yes	Left ICA 63%, Right ICA 65%
CEA13	72	Male	No	Right ICA 91%
ICSS2	55	Female	No	Left ICA 95%, Right ICA 80%
ICSS3	70	Male	Yes	Left ICA 99%

Each optimized configuration, encoded by LMA locations, was integrated with its peripheral vasculature to form a complete vascular graph. LMAs were inserted as edges connecting nodes defined by their six-element labels. Computed flow and pressure values were mapped onto this graph. LMAs were then grouped into seven anatomical pathways based on the vascular regions of their connected nodes. Net collateral flow per path was calculated by summing flow values of all LMAs assigned to it, respecting flow direction. This was repeated for all 200 configurations, producing distributions of net collateral flow per pathway. Configurations are considered valid if the simulated distal flows matched SPECT measurements within 5% for each vascular region:

19 $$\begin{aligned} \left| Q_{\text {MRI}, m} + Q_{\text {in}, m} - Q_{\text {out}, m} - Q_{\text {SP}, m} \right| \le 0.05 \cdot \left| Q_{\text {SP}, m} \right| , \quad \forall m \in \{1, 2, \dots , M\} \end{aligned}.$$

Here, $Q_{\textrm{MRI}, m}$ is the 4D Flow MRI-derived inflow, $Q_{\textrm{in}, m}$ and $Q_{\textrm{out}, m}$ are collateral LMA inflow and outflow, and $Q_{\textrm{SP}, m}$ is the SPECT-measured distal flow. This criterion was met across all regions and cases.

**Fig. 5:**
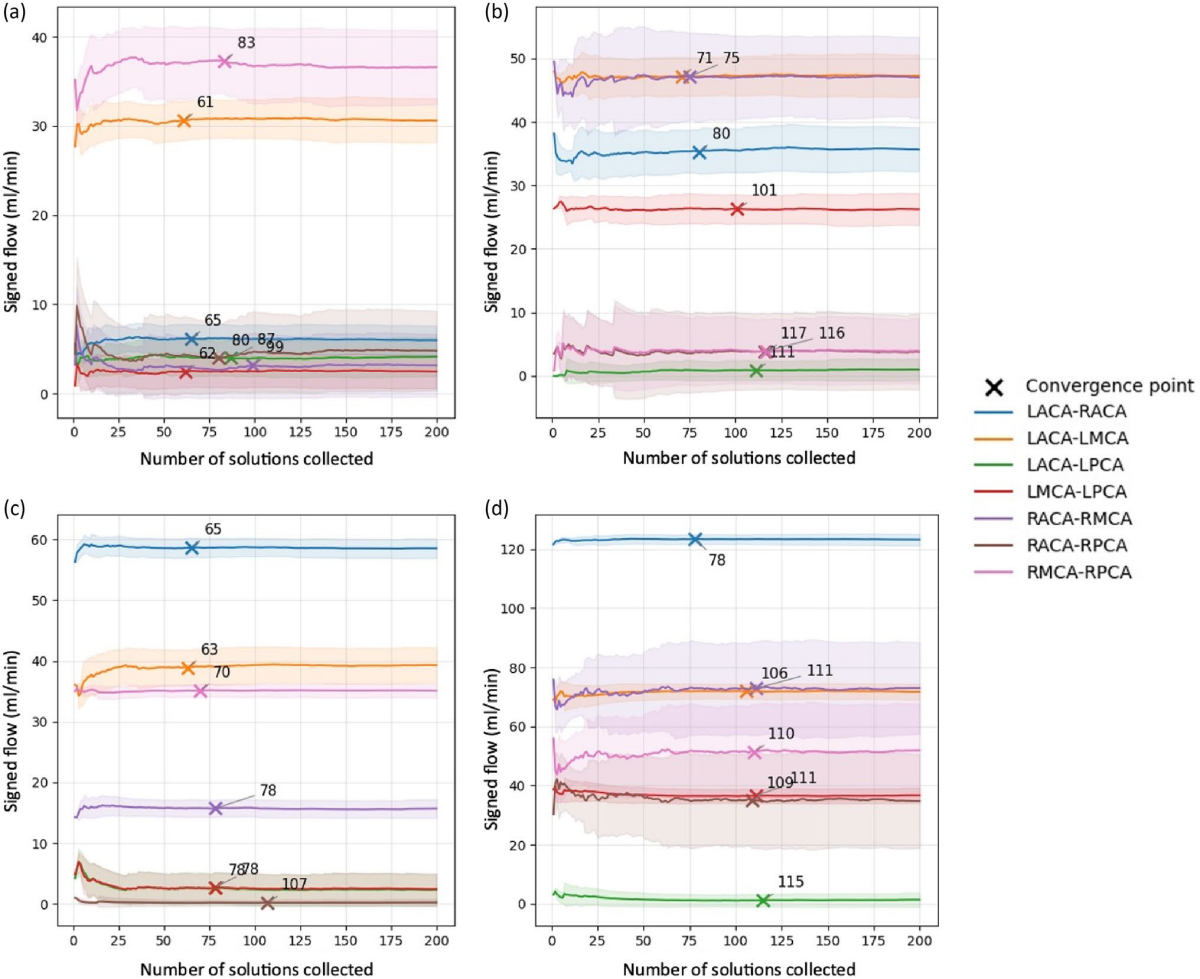
Convergence analysis of ensemble size for **a** CEA12, **b** ICSS2, **c** CEA13, and **d** ICSS3. For each inter-territory pathway, the lines depict the running mean of the signed LMA-mediated flow rate, and the shaded bands represent the corresponding running standard deviation. All pathways reach stable values before $N_{\text {sol}} = 200$, confirming that the selected ensemble size adequately samples the functional network behavior.

In addition, to assess whether the chosen sample size of $N_{\text {sol}} = 200$ adequately captures the functional behavior of the network ensemble, we performed a sensitivity analysis on the evolution of flow-redistribution statistics as additional solution sets were included. For each pathway between cortical regions, we computed the running mean and running standard deviation of the absolute LMA-mediated flow rate as a function of the number of accumulated solutions. Convergence was assessed using a successive-window stability criterion: the running mean and running standard deviation were considered stable once the differences between successive windows of 30 solutions fell below a relative tolerance of 5% of a robust baseline (defined as the median absolute value of all accumulated solutions up to that point).

As shown in Fig. 5, across all vascular pathways, the running mean and standard deviation stabilized well before reaching $N_{\text {sol}} = 200$. Increasing the number of collected solutions did not change the distributional shape of flow rates, which indicates that the functional behavior of the networks is adequately sampled and that additional solution sets would not materially alter the inferred flow-redistribution patterns. Thus, $N_{\text {sol}} = 200$ provides sufficient coverage for the purposes of this study.

For each patient case, the ensemble of $N_{\text {sol}} = 200$ optimization outcomes is analyzed to assess the degree of functional similarity among solutions. Functional similarity is defined in terms of the pattern of flow redistribution between cortical-region pairs. To quantify this, the distribution of flow rate along each collateral pathway is computed across all solutions and assessed for unimodality using Hartigan’s dip test. Across the four patient cases, $27/28$ pathways exhibit unimodal and approximately normal flow distributions, indicating a consistent and reproducible mode of collateral redistribution across the ensemble. The only multimodal case—the RPCA$\rightarrow$RACA pathway in patient CEA12 (Dip statistic $= 0.050$, $p = 0.001$)—arises from the presence of a pericallosal PCA–ACA anastomosis. Because the baseline flow in this pathway is small, intermittent recruitment of this anastomosis introduces a discrete shift in the redistribution pattern, yielding two distinct modes. Overall, the predominance of unimodal pathways demonstrates that the ensemble of $200$ solutions converges toward a coherent functional mode of collateral flow redistribution.

We analyze LMA-mediated collateral flow distributions across seven defined inter-regional pathways. For each case, the net flow per pathway was computed across 200 independently optimized configurations, and the mean and standard deviation are summarized in Table 5. CEA12 and ICSS2, which feature bilateral stenoses, generally exhibit smaller inter-hemispheric flows compared with single-stenosis cases. ICSS3 shows the largest net flow along the LACA–RACA path, consistent with its severe unilateral stenosis and corresponding hemispheric perfusion imbalance. PCA–ACA and MCA–PCA pathways generally carry lower flow, with the exception of the right PCA–ACA path in ICSS3, where a persistent pericallosal anastomosis contributes a substantial fraction of flow.

**Table 5:** Signed flow rate statistics (mean ± standard deviation) for each inter-regional pathway across cases

**Path**	**CEA12 (ml/min)**	**CEA13 (ml/min)**	**ICSS2 (ml/min)**	**ICSS3 (ml/min)**
LACA–RACA	5.98 ± 1.64	58.53 ± 1.58	$-35.68 \pm 3.44$	$-123.17 \pm 1.96$
LACA–LMCA	$-30.61 \pm 2.44$	$-39.30 \pm 2.90$	47.28 $\pm 3.34$	71.73 $\pm 2.70$
LACA–LPCA	$-4.12 \pm 2.20$	$-2.28 \pm 2.72$	0.24 $\pm 1.94$	1.28 $\pm 2.39$
LMCA–LPCA	$-2.21 \pm 2.17$	2.24 $\pm 2.64$	$-26.27 \pm 2.51$	$-36.69 \pm 2.55$
RACA–RMCA	2.58 ± 3.94	15.69 $\pm 1.55$	$-47.01 \pm 6.38$	$-73.03 \pm 15.48$
RACA–RPCA	$-4.79 \pm 4.46$	0.04 $\pm 0.59$	$-3.77 \pm 5.97$	$-34.73 \pm 15.84$
RMCA–RPCA	$-36.58 \pm 4.13$	$-35.07 \pm 1.12$	2.99 $\pm 5.74$	$-51.90 \pm 15.79$

**Fig. 6:**
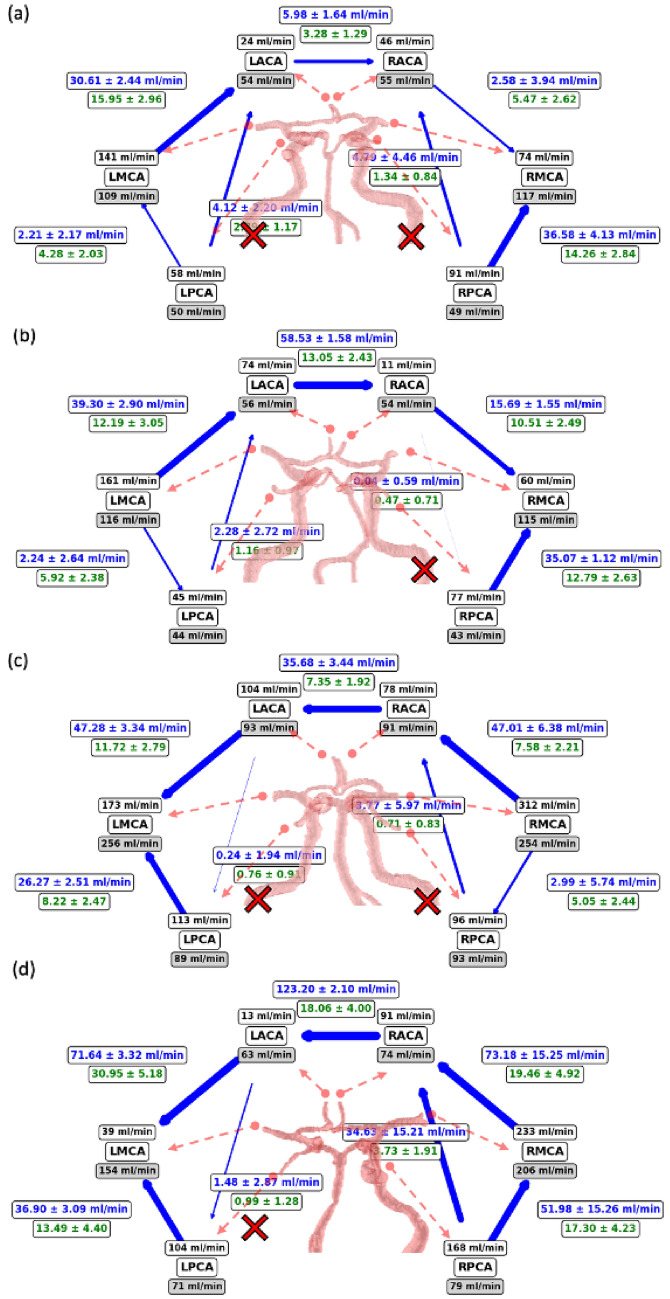
Flow rate redistribution via LMAs for **a** CEA12, **b** CEA13, **c** ICSS2, and **d** ICSS3, based on 200 LMA configurations. The semi-transparent, patient-specific CoW model is shown at the center. Red crosses mark ICA stenoses. Blue arrows and numbers indicate mean flow rates; green numbers show mean LMA counts. Plus-minus values denote standard deviation. The left ICA in **d** is missing due to near-occlusion on CT-angiography images.

Beyond pathway-level LMA configurations, the model revealed emergent global redistribution patterns. Figure 6 reformat Table 5 to highlight flow direction and magnitude, supporting cross-case comparison. For each region, 4D Flow MRI-derived inflow is displayed above, and SPECT-based distal flow below. Mean net LMA-mediated flow for each pathway (blue) and average LMA count (green) are shown, with standard deviations indicating anatomical and optimization variability.

**Fig. 7:**
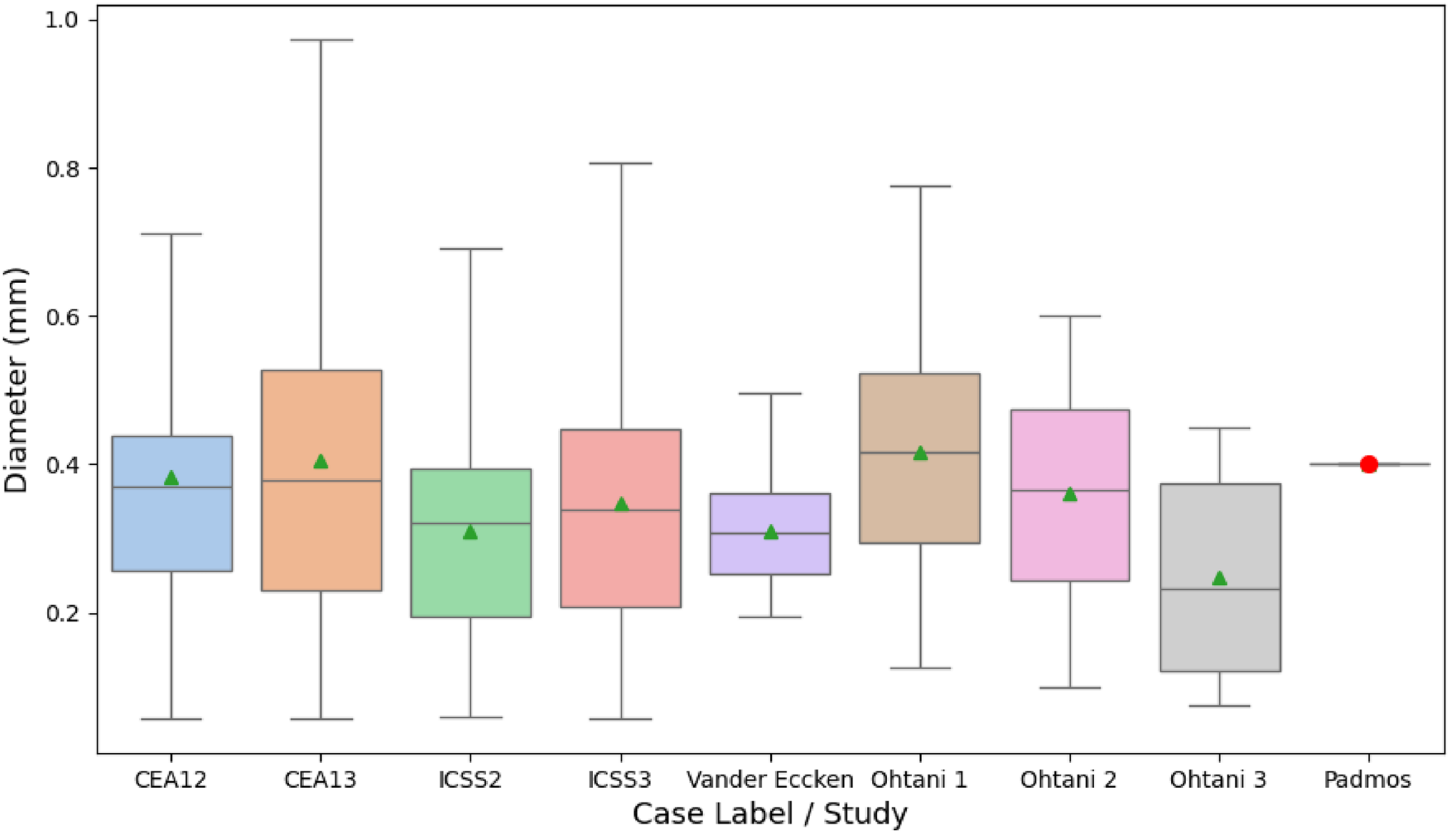
Diameter distribution of LMAs in the four patient cases used in this study, compared with literature values: cadaver data from Vander Eecken and Adams [9], modeling results from Ohtani et al. [22], and from Padmos et al. [15]. Ohtani et al. presented three configurations of vasculature with varying range of diameter for arterioles available for LMA connections, shown as Ohtani 1, 2, and 3. Padmos et al. assumed a constant LMA diameter of 0.4 mm.

LMA networks generated by the synthetic vasculature model align with prior anatomical and computational studies. Figure 7 shows average LMA diameters across cases, compared with Padmos et al. [15] and cadaver data from Vander Eecken and Adams [9]. While our model produces a wider diameter range than Vander Eecken’s (medians from 0.05-−0.7 mm), it more closely matches Ohtani et al. [22].

Quantitative data on LMA counts, particularly under ischemia, are limited. Vander Eecken and Adams [9] observed 8–14 LMAs in cadaver brains, without physiological context. Ohtani et al. [22] showed that in MCA stenosis, collateral flow recovery plateaued near 30 LMAs along the ACA–MCA route, measured by a recovery ratio. Direct comparison is difficult: our model lacks baseline flow data for recovery ratio computation and includes multiple inter-territory routes, unlike their single-route setup. Still, our LMA counts in ICSS3—featuring near-occlusion of the right ICA—are comparable in scale and only reach the plateau in one pathway. Zhao et al. [40] similarly found that increasing LMAs from 5 to 11 significantly improved MCA oxygen saturation during occlusion. Like Ohtani et al., they studied one path, but our inter-regional LMA counts (typically 5–15 except in ICSS3) are consistent with their findings.

**Fig. 8:**
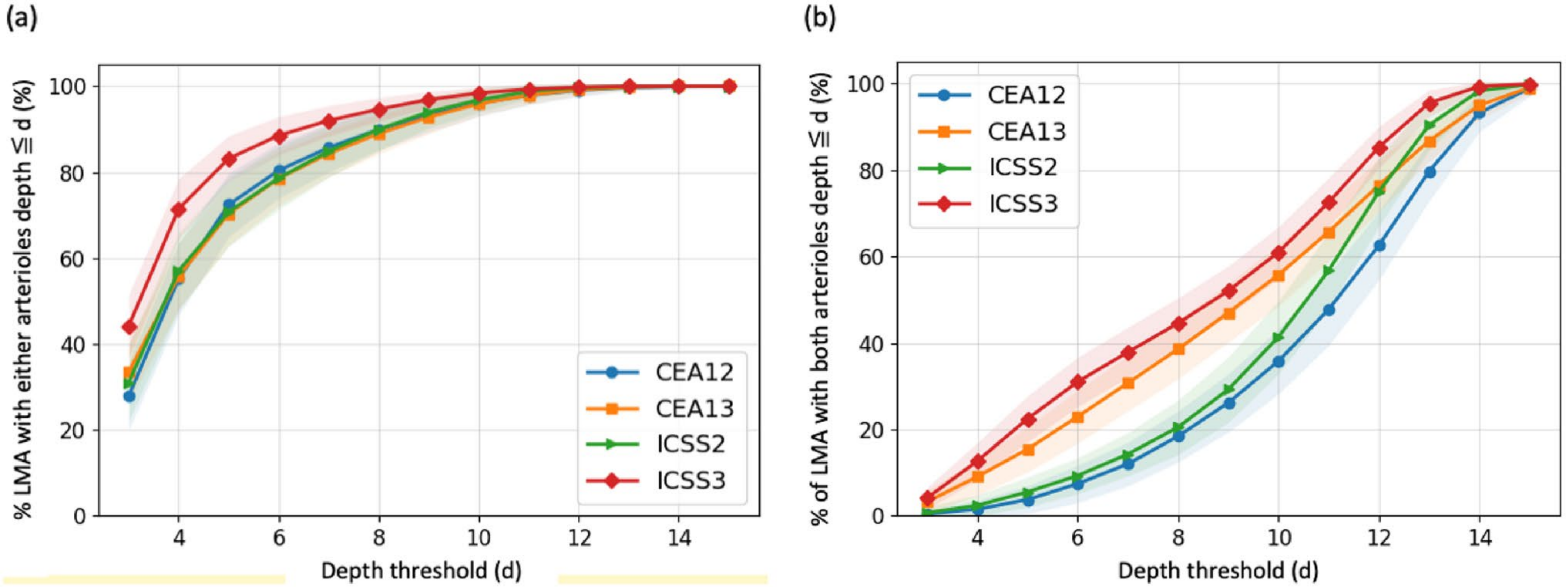
Cumulative proportion of LMAs as a function of the bifurcation depth of their two connection arterioles, averaged over 200 independently optimized configurations. **a** Proportion of LMAs for which at least one connection arteriole is shallower than depth *d*. **b** Proportion for which both connection arterioles are shallower than depth *d*. Solid curves denote the mean across configurations; shaded regions denote one standard deviation.

For each LMA in each of the 200 independently optimized configurations per patient case, the bifurcation depths of its two connection arterioles were recorded. Using these depths, we computed cumulative proportions describing how frequently LMAs draw from arterioles shallower than a given threshold. Two complementary curves were evaluated: Fig. 8a quantifying LMAs in which at least one connection arteriole lies within the depth threshold, and Fig. 8b quantifying LMAs in which both connection arterioles lie within the threshold.

Across all cases, the curves in Fig. 8a rise rapidly and saturate early. By depth 5, the cumulative proportion reaches approximately 70% in CEA12, CEA13, and ICSS2, and exceeds 80% in ICSS3. This consistent pattern indicates that most LMAs involve at least one relatively proximal connection.

Figure 8b reveals stronger case-to-case differences. CEA13 and ICSS3 increase more linearly with depth, reflecting a larger fraction of LMAs whose both connections are proximal. In contrast, CEA12 and ICSS2 exhibit flatter initial portions that rise more noticeably only at deeper thresholds, indicating fewer proximal–proximal pairings and more proximal–distal pairings. ICSS3 shows the most pronounced early growth. Together these curves outline two structurally distinct patterns: a shared tendency to incorporate at least one proximal arteriole, and a case-specific modulation of how frequently pairs of proximal arterioles are selected.

Simulation runtime varied across cases, primarily reflecting the number of LMAs required for effective collateral redistribution. As shown in Table 6, more LMAs increased both solution space complexity and computational demand. Runtimes grew superlinearly with LMA count due to the heavier use of length adjustment and mutation, which are more costly than crossover. Cases requiring greater redistribution naturally exhibited longer runtimes. ICSS3, with near-occlusive ICA, exemplifies the upper bound of current computational feasibility. While further optimization (e.g., parameter tuning) is possible, the current implementation remains effective.

**Table 6:** Computational performance across cases

**Case ID**	**Time per set (s)**	**Average # of LMAs**	**% of Total CBF Redistributed**
CEA12	72	47	20.0%
CEA13	81	56	35.8%
ICSS2	63	41	18.6%
ICSS3	396	104	60.6%

## Discussion

Flow spread across configurations anatomical and functional collateral routing flexibility. For example, in ICSS3’s healthy right hemisphere, collateral flow toward the left hemisphere may proceed directly via PCA–ACA or indirectly via PCA–MCA then MCA–ACA, depending on vascular structure. Conversely, flow spread is narrow in ICSS3’s left hemisphere and other cases due to limited ACA–PCA connectivity restricting collateral options. Absence of a full roundabout connection among ACA, MCA, and PCA, which is only present in ICSS3’s right hemisphere, causes redistribution to be constrained to a single dominant path. Alternative routes are either absent or negligible, producing consistent net flow and narrower distributions.

As expected, LMA count alone does not predict flow redistribution, as efficiency also depends on pressure gradients between connected regions. While LMA radius is constrained and stable due to network topology, pressure differences vary and critically determine flow.

LMA-mediated redistribution strongly correlated with ICA stenosis severity and asymmetry. Higher-grade stenosis increased overall collateral flow, evident when comparing ICSS3 to CEA13 (unilateral cases) and ICSS2 to CEA12 (bilateral cases). This is consistent with pressure-driven flow: severe ICA narrowing causes distal pressure drops, particularly in anterior regions, drawing blood from posterior areas via routes like PCA–MCA. Additionally, single-sided stenosis (ICSS3, CEA13) produced greater inter-hemispheric flow than bilateral cases due to larger pressure asymmetry, activating multiple vascular pathways. In bilateral stenosis with similar stenosis ratio in both ICAs, symmetric hypo-perfusion reduced gradients and limited redistribution.

Clinical outcomes, however, did not correlate directly with stenosis severity. For instance, CEA12—symptomatic with limb and visual deficits—had modest stenosis but notably low total CBF. This may stem from high microvascular resistance or limited collateral capacity. Supporting data indicate left MCA territory blood stealing during acetazolamide challenge, suggesting depleted cerebrovascular reserve. Such hemodynamic exhaustion aligns with occlusive disease patterns [41]. ICSS2, despite bilateral high-grade stenosis (95%, 80%), maintained near-normal flow and no hypo-perfusion signs. Although acetazolamide data were unavailable, clinicians observed low infarction and hyperperfusion risk, indicating effective compensation. These case comparisons illustrate the complex and case-dependent relationship between stenosis severity, CBF redistribution, and clinical outcome. They highlight the functional importance of LMA-mediated collateral pathways in modulating hemodynamic response to stenosis, and, more importantly, suggest that assessing flow redistribution—rather than stenosis severity alone—may offer a more accurate reflection of patient status.

The validity of the proposed model is supported by its mechanistic and physiologically interpretable behavior. LMA diameters are consistent across cases and physiological states, reflecting underlying structural constraints rather than arbitrary optimization outcomes. This stability arises because LMAs connect level 3 arterioles, whose sizes are constrained by upstream level 2 arteries; as level 2 diameters fall within a narrow physiological range, LMA sizes naturally follow, independent of the number or specific placement of LMAs.

Moreover, LMA-mediated redistribution closely reflects both the severity and symmetry of ICA stenosis. In cases of higher-grade stenosis, overall collateral flow increases, as seen when comparing ICSS3 to CEA13 (unilateral cases) and ICSS2 to CEA12 (bilateral cases), consistent with pressure-driven dynamics: severe narrowing of the ICA lowers distal pressures, particularly in anterior regions, drawing blood from posterior territories via pathways such as the PCA–MCA. Furthermore, asymmetry in stenosis influences inter-hemispheric redistribution. Single-sided stenosis (ICSS3, CEA13) generates larger pressure differences across hemispheres, enhancing inter-hemispheric flow. By contrast, bilateral stenosis with similar severity in both ICAs produces more symmetric hypo-perfusion, reducing pressure gradients and limiting redistribution.

Analysis on connection depth of LMAs offers further evidence that the model’s optimization behavior is physiologically grounded. A consistent feature across all cases is the rapid initial rise in Fig. 8a, reflecting the widespread use of at least one proximal connection arteriole. This behavior follows directly from the basic vascular physics of cortical trees. As arterioles extend distally within a vascular territory, both their radii and their pressures decrease: radii decline through hierarchical bifurcation following Murray’s law, and pressures fall due to cumulative viscous losses along the branching path. Because each LMA inherits a radius equal to the average of its two connection arterioles, pairing two proximal arterioles maximizes caliber and minimizes LMA resistance. Conversely, combining a proximal with a distal arteriole yields the largest pressure difference between territories, which strongly drives collateral flow. These represent two extremal hemodynamic strategies—radius maximization and pressure-drop maximization—while distal–distal pairings offer neither advantage and thus appear less frequently. The fact that the rapid initial rise is a shared feature across cases indicates that this fundamental trade-off, not case-specific structure, governs the model’s behavior.

Figure 8b isolates how subject-specific hemodynamics influence which of the two extremal strategies is favored. CEA13 and ICSS3, both single-stenosis cases, show more linear increases, indicating a higher prevalence of proximal–proximal connections. Their elevated interterritorial pressure gradients allow the model to exploit larger LMA radii without sacrificing the driving pressure required for collateral flow. ICSS3, with its near-occlusion, shows this tendency most strongly. In contrast, the bilateral-stenosis cases (CEA12 and ICSS2) exhibit comparatively low early growth, indicating fewer proximal–proximal pairings. This pattern reflects a different hemodynamic compromise: with smaller interterritorial pressure gradients, the model cannot rely on a large driving pressure to push flow across a low-resistance (large-radius) LMA. To recover sufficient pressure drop across the collateral, the model therefore more often selects asymmetric pairings that combine a proximal and a distal arteriole. Such proximal–distal connections increase the pressure difference between the LMA termini, at the cost of reducing the LMA’s average radius and therefore increasing its resistance, but the larger pressure gradient compensates, enabling effective collateral flow.

Our model demonstrates distinct strengths in terms of clinical applicability, particularly in scenarios where detailed anatomical data are limited or unavailable. In practice, detailed anatomical data are often unavailable due to imaging limitations. Even when available, segmentation is error-prone and resolution-limited (0.3-−0.5 mm), making precise modeling of small vessels difficult. Despite advances in deep-learning-based super-resolution techniques [42], poor-quality images still limit accuracy.

Given these challenges, our model accounts for uncertainty in vasculature by representing the vascular network probabilistically. Rather than relying on a single deterministic anatomical configuration, the model considers a range of possible vascular structures. This probabilistic approach enables the simulation of vascular networks without requiring precise, patient-specific data for small arteries like level 2 arteries. It provides a safer approach when the actual structure is unknown or uncertain, ensuring robustness in the model’s predictions regardless of data availability.

A key strength of our model lies in its modular composition, which offers inherent flexibility in accommodating different levels of anatomical data. The multi-level generative framework allows for the integration of anatomical data at various stages of modeling, depending on data availability. For instance, if detailed imaging data for level 2 arteries becomes available, the model can transition from a probabilistic to a more deterministic representation of these arteries. Conversely, when detailed imaging data are unavailable or uncertain, the model can still simulate vascular networks probabilistically, preserving the core functionality of collateral recruitment and LMA dynamics. This modularity makes the model adaptable, facilitating both uncertainty analysis and anatomically detailed simulations. Although less spatially detailed than CCO-based models, our framework looks to prioritize flexibility and clinical feasibility. It provides a platform for exploring hemodynamics and LMA recruitment with limited data, offering practical value in real-world settings.

### Limitations

Several limitations temper the interpretation of this work. First, the model assumes a fixed pial surface pressure across cortical territories. In practice, pial surface pressures are influenced by cerebral autoregulation, whereby arteriolar diameter is actively modulated in response to changes in perfusion pressure. Autoregulatory responses have been shown to vary significantly across patients due to differences in vasoconstrictive capacity [43]. Because LMA-mediated redistribution depends on pressure gradients between connected cortical territories, such variability in pial pressures may influence both the magnitude and spatial pattern of collateral recruitment.

Secondly, cortical territories are represented as idealized rectangular segments, which reduces the spatial fidelity of LMA placement. This simplification may permit connections that would be geometrically infeasible within a fully resolved pial network. More anatomically accurate, patient-specific pial networks can be generated using constructive algorithms, such as that proposed by Ii et al. [21]. However, the computational cost of such approaches is incompatible with the present workflow, which requires the evaluation of thousands of candidate vascular structures under uncertainty. Nevertheless, these models could serve as an anatomical atlas for comparison in future work. Given the relatively coarse spatial resolution of the SPECT data, increased geometric fidelity would be unlikely to yield proportionate gains in prediction accuracy within the scope of this study.

Thirdly, the optimization framework is constrained by physiological principles, yet the absence of ground-truth LMA maps limits independent validation and precludes the exclusion of subtle overfitting. Direct imaging of LMAs, for example via high-resolution angiography [5], could in future provide empirical validation and inform more accurate patient-specific modeling.

Finally, the synthetic vascular networks are generated from statistics reported in the literature for healthy subjects and may not capture disease-specific microvasculature. As a result, pathologies such as Moyamoya disease, which markedly alter peripheral arteriolar caliber and vascular topology [44], could produce collateral patterns fundamentally different from those represented in the present model. Similarly, patient-specific peripheral vascular anatomy cannot be fully resolved without additional imaging, limiting the generalizability of the inferred LMA distributions. These limitations indicate potential directions for future studies, should richer anatomical or functional data become available.

## Conclusion

In conclusion, the developed model, with its multi-level generative structure, enables a probabilistic analysis of the role and functionality of LMAs in a patient-specific context, with modeled LMAs agreeing in size and quantity with existing literature. It successfully identifies solutions within physiological constraints across four distinct clinical scenarios—ranging from mild bilateral stenosis to severe, asymmetric, and symptomatic unilateral stenosis. This robustness suggests that the LMA model is not overly constrained by the physiological assumptions it incorporates, allowing it to adapt to varying flow-redistribution demands.

## Data Availability

The datasets generated and analyzed during the current study are available from the corresponding author on reasonable request.
